# Dynamic Navigated “Sandwich” Technique: A Novel Surgical Approach for Safe Osteotomies in the Rehabilitation of an Atrophic Posterior Mandible: A Case Report

**DOI:** 10.3390/mps4020034

**Published:** 2021-05-16

**Authors:** Pietro Felice, Lorenzo Bonifazi, Maryia Karaban, Cesare Berti, Gerardo Pellegrino, Carlo Barausse

**Affiliations:** 1 Oral Surgery, Department of Biomedical and Neuromotor Sciences, University of Bologna, 40125 Bologna, Italy; lorenzo.bonifazi@studio.unibo.it (L.B.); maryia.karaban@studio.unibo.it (M.K.); cesare.berti@studio.unibo.it (C.B.); gerardo.pellegrino2@unibo.it (G.P.); carlo.barausse2@unibo.it (C.B.); 2 Postgraduate School of Oral Surgery, University of Modena and Reggio Emilia, 41124 Modena, Italy

**Keywords:** atrophic posterior mandible, inlay bone graft, dynamic navigation, piezosurgery, safe osteotomies

## Abstract

A 56-year-old female patient with vertical atrophy of the right posterior mandible was treated adopting an interpositional bone block approach using a cancellous heterologous bone block. Osteotomies of the patient’s mandible were performed with the help of dynamic computer-assisted surgery using virtual anatomical patient information obtained from a cone beam computed tomography (CBCT). The use of the dynamic computer-assisted surgery allowed authors to perform the horizontal osteotomy line as planned preoperatively on the CBCT virtual reconstruction, trying to minimize the risks of the inlay technique. No neurological complications were observed after surgery. The inlay technique could benefit from the aid of dynamic navigation technologies in posterior atrophic mandibles, increasing the reproducibility of the technique. A likely safer method for performing osteotomies with the “sandwich” technique in the posterior atrophic mandible is reported.

## 1. Introduction

The posterior mandible area is probably the most complex area to rehabilitate with a fixed implant solution due to the proximity of the inferior alveolar nerve. Different procedures have been proposed to achieve bone augmentation, including guided bone regeneration (GBR), distraction osteogenesis, and inlay and onlay bone grafting. However, it is difficult to determine which one in particular gives better clinical outcomes than the others, and the number of failures or complications related to all techniques for vertical bone augmentation remains high [1,2].

First proposed by Schettler in 1976, the inlay technique allows for the obtaining of positive outcomes in vertical bone augmentations thanks to favorable vascular supply, and to reduce bone resorption [3,4,5]. The “sandwich” technique, described in the literature for the mandible and for the maxilla, involves the interposition of a bone block in a new space obtained by lifting a bone segment after a horizontal and two vertical osteotomies. In this procedure, it is possible to use different kinds of grafts: autogenous, allogenic, or xenogenic [6]. Nowadays, xenografts show similar outcomes to autografts, but are associated with a less invasive surgery avoiding autogenous bone harvesting. Thus, xenografts are more preferable for both the patient and the clinician [7]. 

To obtain bone segments that can be lifted, a horizontal osteotomy is performed following the inferior alveolar nerve course. It is also fundamental to ensure the correct distance from the alveolar nerve to avoid neurological complications [7]. 

As noted in various studies, the use of navigated surgery could nowadays be considered to be an improvement over freehand, and the same as static guided surgery, as far as precision is concerned in implant placement [8]. The authors’ aim is a safe-guided osteotomy performed with a dynamic computer-assisted surgery system, to split the residual bone thickness between inferior alveolar nerve and alveolar ridge, obtaining the right osteotomized bone segment height and a safe distance from the canal. 

## 2. Materials and Methods

A 56-year-old female in good health was referred requiring a fixed prosthetic rehabilitation of the premolar and molar zone in the posterior right mandible. A preoperatory Cone Beam Computerized Tomography (CBCT) scan was performed to evaluate alveolar ridge residual bone anatomy and to plan implant placement. The evaluation of the images obtained showed a moderate mandibular vertical atrophy, around 7 mm, precluding the insertion of implants of standard length. As a treatment plan, rehabilitation using short implants was proposed to the patient, since this is a posterior non-aesthetic area. However, the patient had high aesthetic expectations and asked for a crown length similar to natural teeth. For this reason, a “sandwich” vertical augmentation procedure was chosen to reconstruct the bone in order to place standard implants. All possible benefits and complications were discussed with the patient and written informed consent was obtained. The clinical case was conducted according to the principles of the Declaration of Helsinki on experimentation involving human subjects. 

**Figure 1:**
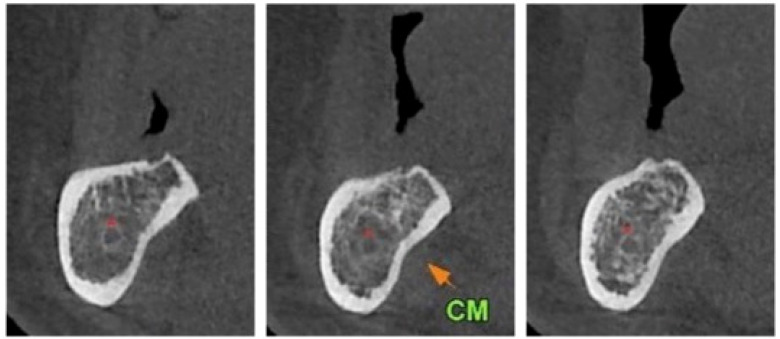
Preoperative Cone Beam Computerized Tomography (CBCT) showing the vertical bone atrophy.

Surgeons chose dynamic navigation as a system that could improve the accuracy and safety of the sandwich technique. A technology called Trace Registration (TR) was used. Instead of a radiographic marker, TR exploits the already existing radiographic landmarks in the scan, such as teeth, abutments, or certain types of artificial crowns. This allows the use of the standard, diagnostic CBCT scan, without the need to introduce an artificial marker into it, therefore, reducing the number of workflow steps and further simplifying the process (Figure 1). TR works through a “Surface Contact Scan” approach. A tracer was used prior to surgery to trace the surface of among three to six residual teeth. For the system’s camera to track the patient’s jaw, an optical tracking tag needs to be fixed to the jaw on which surgery will be performed. This requires a Jaw Tracker (a combination of the optical tag and bendable metal wire) to be connected to one or two teeth in the residual dentition with a light-cured composite resin. By sampling the surface of these teeth with the tracer, a virtual 3D mesh representation of the surface of these landmarks was generated. This 3D mesh was matched with the surface of the traced landmarks by the software, to generate the registration of the CBCT with the physical patient’s jaw, thus achieving the same purpose as the radiopaque stent. The surgeon then verified the registration accuracy by placing the tracer tip on the patient’s teeth from several aspects and comparing the physical location of the tip with its representation on the system’s screen. 

**Figure 2:**
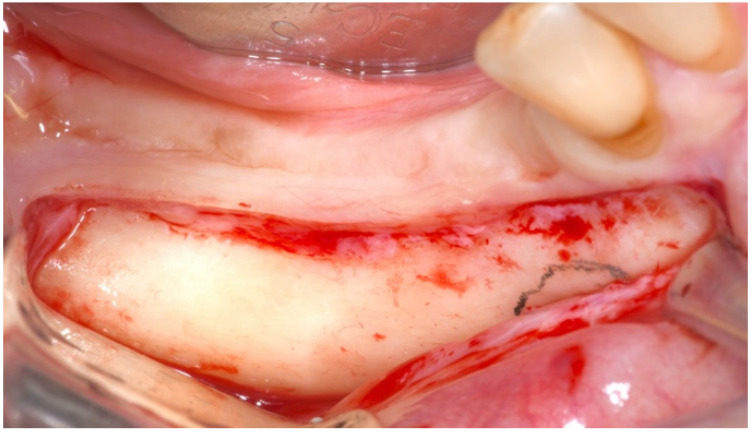
A paracrestal incision was made through the buccal nonkeratinized tissue, respecting the emergence of the mental nerve. A mucoperiosteal flap was then raised.

**Figure 3:**
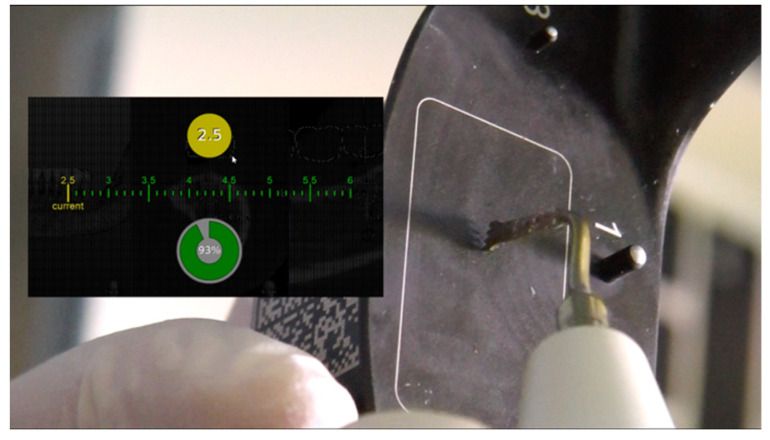
Dynamic navigated “sandwich” technique: tip calibration.

After the described procedures, the surgeon made a paracrestal incision in the buccal aspect and a flap was elevated avoiding tension on the mental nerve (Figure 2). No mucoperiosteal dissection was performed toward the alveolar crest or on the lingual side to preserve adequate blood supply to the bone segment to be osteotomized. The surgical tip of the piezosurgery (Piezosurgery, Mectron, Carasco, Italy) was calibrated, and a second verification of the accuracy was carried out: the surgeon placed the piezo insert tip on the tooth surface, comparing to the on-screen representation (Figure 3).

**Figure 4:**
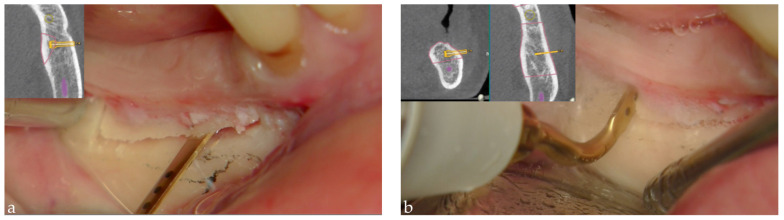
Dynamic navigated “sandwich” technique: (**a**) the piezo-tip was used to obtain the pre-established horizontal osteotomy, approximately 2 mm above the mandibular canal, and (**b**) the distal vertical one. The software, as the surgeon operates, provides an indication of where the piezosurgical insert was located relating to the bone and nerve.

**Figure 5:**
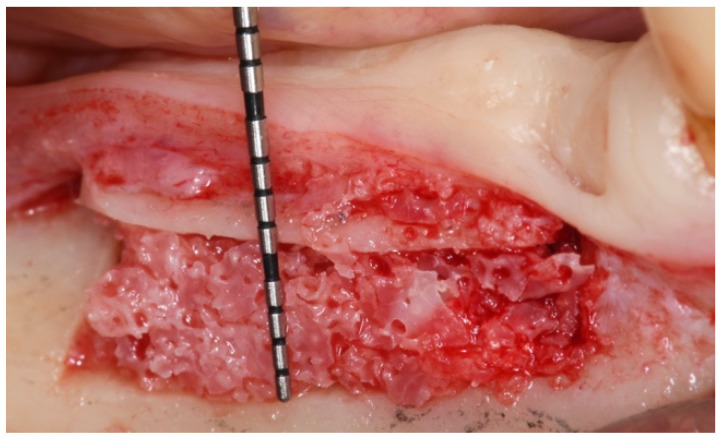
An appropriately shaped cancellous block of heterologous bone was then placed into the space obtained between the basal bone and the osteotomized segment.

Then, the same piezo-tip was used to obtain the pre-established horizontal osteotomy, approximately 2 mm above the mandibular canal, and for the vertical ones, the first 2 mm distal to the last residual tooth and the second according to the rehabilitative plan. As the surgeon operates, the software provides an indication of where the piezosurgical insert was located relating to the bone and nerve (Figure 4). An appropriately shaped cancellous block of heterologous bone (Sp-Block, Osteobiol, Tecnoss, Giaveno, Italy) was then placed into the space obtained between the basal bone and the raised osteotomized segment (Figure 5). A resorbable collagen membrane was applied above the buccal surface to protect the surgical site. The flaps were sutured with Vicryl 5.0. The patient was then prescribed a fluoroquinolone antibiotic (moxifloxacin) twice a day for 5 days combined with azithromycin twice a day for 3 days. She was instructed to take the first tablet the night before surgery and the second one 2 h before surgery. Moreover, betamethasone was administered just after the surgery (8 mg, then tapering the daily dose) and 600 mg of ibuprofen twice a day, to be taken with meals, as long as required. Postsurgical instructions were delivered, including a soft diet for 2 weeks and appropriate oral hygiene with twice a day rinsing with 0.2% chlorhexidine gluconate mouthwash. The patient was clinically checked two weeks after surgery and at one month. No neurosensory alterations or other complications were recorded. 

## 3. Discussion

Bone augmentation techniques in vertical defects of the posterior mandible are very complex treatments with several possible complications, but it is the only option to get an aesthetic prosthesis of adequate crown length in patients with high aesthetic needs [2]. 

There is no regeneration technique that has been proven to be able to provide better results than the others [1]. The GBR could be a suitable option, but the authors of this article suggest, in the case of a minimum of 5 mm of residual bone height, the interpositional block bone graft to vertically augment the atrophic posterior mandible as this approach seems to guarantee a greater vascular supply, coming from the lingual periosteum and the residual bone to the internal graft; it also allows for the use of the native basal bone, which should be less prone to resorption around the implant head. Moreover, the study of Felice et al. (2017) reported that an implant survival rate in bone augmented with the inlay technique after a 4.2-year mean follow-up ranges from 91.1 to 96.0%, with a favorable peri-implant marginal bone loss (1.37 mm after 7 years loading) and bone height increase (5.75 mm) using xenografts [9].

Another option is short implants, an alternative that is becoming increasingly used, which allows for less invasive surgery and a shorter rehabilitation period but do not guarantee the aesthetic length of the final rehabilitation [10,11,12,13,14,15,16,17].

Considering all these aspects, for the first experience of the authors’ work with dynamic navigation in combination with the sandwich technique, a patient who needed good aesthetics was chosen. A clinical case with a level of atrophy that was guaranteed to work safely was preferred, thus avoiding borderline situations.

It has to be considered that the inlay technique can present multiple risks of complications such as paresthesia of the inferior alveolar nerve and bone fracture, but it provides good results in terms of survival implant rates and peri-implant bone loss [18]. This could be related to the fact that the cranial osteotomized bone segment is completely made of native bone. 

In a study by Felice et al. (2014), the use of a static surgical template to guide the osteotomy, minimizing the risks mentioned above, showing positive results, and paving the way for further research is described [18]. The necessary use of a surgical template, however, entails disadvantages from the point of view of occupied volume, therefore in intraoperative visibility, and of lack of versatility, in the case of necessary changes to the previous planning.

With regards to the use of the piezoelectric device in oral surgery, it has seen to be a valid alternative to rotary instruments, reducing surgical trauma and, therefore, the risk of neurological complications [19]. 

Given the growing number of studies showing the accuracy of dynamic navigation systems in dental implants, which appears to be similar to that of static ones but with greater versatility, in this case report, the authors chose to combine this digital dentistry technology with the inlay technique [20,21,22,23,24,25]. 

Starting from a preoperative CBCT, it is possible to plan the horizontal and vertical osteotomies, so that, once the tip of the piezoelectric handpiece is calibrated (Figure 3), its position can be known moment by moment during the surgery in relation to what was planned before on the software. This aspect could be also useful in reducing the possible lingual periosteum damaging, which can lead to a graft exposure.

While for experienced surgeons it is already possible to approach freehand even when the nerve is only 2 mm above, the software could provide an indication of where the piezo tip is located relating to the bone and nerve as the surgeon operates (Figure 4A,B).

Therefore, according to the authors, the possibility of preoperative planning could allow not only to reduce time and uncertainties during the surgical procedure, but also to safely position the horizontal osteotomy next to the nerve, obtaining the maximum thickness of the bone segment, and to preserve the lingual periosteum from potential injury. Moreover, unlike guided static surgery, the use of the intraoperative navigator allows to modify the cut, if the anatomy or the different clinical circumstances requires it, and to have an excellent visibility of the operative field, not involving any physical template. 

The use of the intraoperative navigation also includes some disadvantages, such as the need for correct preoperative planning, and, therefore, for a CBCT performed with specific intraoral markers, and an increase in surgical times. All this together with the technological investment, can lead to an increase in the cost for the patient.

The accuracy of dynamic implant navigation referring to implant placement is reported in the literature, with the mean entry point and apex deviation as well as overall angle discrepancy measured (0.59 mm, 0.85 mm, and 1.98 degrees, respectively) [13]. These results found in the implant positioning can be encouraging, but it has to be considered that there is a lack of data for the precision of this technology in absolute terms, therefore, additional randomized controlled trials (RCTs) aimed at evaluating the navigation in oral surgery are requested. 

The aim of the case report was to describe how new technologies such as dynamic navigation could bring surgical advantages to the clinician. The interpositional bone block technique is already well-described, however, the main limitation of this approach could be related to the safety of the horizontal osteotomy. With this new navigated option, the authors preliminarily tested the possible upgrade in a safer and more confident surgery. This was just an initial case to evaluate the feasibility of this new approach, which is surgically centered. The main aspect the authors wanted to test was about a healthy post-operative course without nerve impairment.

The clinical limits of the technique could be related to the initial cost of the navigator, to the required amount of space for the intra-oral tools, and to the learning curve, which could initially make surgical times longer.

It is also important to take into consideration that, in any case, this is a complex surgical technique requiring experience to proper manage the technology limitations. Moreover, proper hygienic care and control plays, in every phase, a fundamental role [26,27].

Future research directions [28] could be related to the better understanding of the biologic principles associated with this technique with the help of innovative diagnostic techniques such as microtomography and peripheral quantitative computed tomography (pQCT) [29,30,31].

## 4. Conclusions

Being aware of the multiple limits of a case report, the described case could be an example of the usefulness and versatility of the dynamic computer-assisted surgery. No neurological complications were observed, with an uneventful postoperative course. Therefore, the Inlay Technique could benefit from the aid of dynamic navigation technologies in posterior atrophic mandibles, attempting to reduce possible intraoperative surgical complications. However, this is only an initial clinical case that needs further and more representative RCTs to better define the advantages of this procedure and to compare it with freehand or static computer-guided inlay techniques.

## References

[1] Chiapasco M., Casentini P., Zaniboni M. (2009). Bone Augmentation Procedures in Implant Dentistry. Int. J. Oral Maxillofac. Implant..

[2] Esposito M., Grusovin M.G., Felice P., Karatzopoulos G., Worthington H.V., Coulthard P. (2009). The efficacy of horizontal and vertical bone augmentation procedures for dental implants-A Cochrane systematic review. Eur. J. Oral Implantol..

[3] Schettler D. (1976). Sandwich-technic with cartilage transplant for raising the alveolar process in the lower jaw. Fortschr. Kiefer Gesichtschir..

[4] Stoelinga P.J.W., Tidemann J.S., Berger H., de Koonen A. (1978). Interpositional bone graft augmentation of the atrophic mandible. J. Oral Surg..

[5] Zins J.E., Kusiak J.F., Whitaker L.A. (1984). The influence of the recipient site on bone grafts to the face. Plast. Reconstr. Surg..

[6] Misch C.M., Misch C.E., Resnik R.R., Ismail Y.H. (1992). Reconstruction of maxillary alveolar defects with mandibular symphysis grafts for dental implants: A preliminary procedural report. Int. J. Oral Maxillofac. Implant..

[7] Felice P., Marchetti C., Iezzi G., Piattelli A., Worthington H., Pellegrino G., Esposito M. (2009). Vertical ridge augmentation of the atrophic posterior mandible with interpositional bloc grafts: Bone from the iliac crest vs. bovine anorganic bone. Clinical and histological results up to one year after loading from a randomized-controlled clinical trial. Clin. Oral Implant. Res..

[8] Block M.S., Emery R.W., Lank K., Ryan J. (2017). Implant Placement Accuracy Using Dynamic Navigation. Int. J. Oral Maxillofac. Implant..

[9] Felice P., Barausse C., Zucchelli G., Piattelli M., Ippolito D.R. (2017). Interpositional Augmentation Technique in the Treatment of Posterior Mandibular Atrophies: A Retrospective Study Comparing 129 Autogenous and Heterologous Bone Blocks with 2 to 7 Years Follow-Up. Int. J. Period. Rest. Dent..

[10] Esposito M., Buti J., Barausse C., Gasparro R., Sammartino G., Felice P. (2019). Short implants versus longer implants in vertically augmented atrophic mandibles: A systematic review of randomised controlled trials with a 5-year post- loading follow-up. Int. J. Oral Implantol..

[11] Felice P., Barausse C., Pistilli R., Ippolito D.R., Esposito M. (2018). Short implants versus longer implants in vertically augmented posterior mandibles: Result at 8 years after loading from a randomised controlled trial. Eur. J. Oral Implantol..

[12] Barausse C., Maranesi T., Pistilli R., Felice P. (2017). Short implants: An alternative to bone augmentation in atrophic patients. Dental. Cadmos..

[13] Bolle C., Felice P., Barausse C., Pistilli V., Trullenque-Eriksson A., Esposito M. (2018). 4 mm long vs longer implants in augmented bone in posterior atrophic jaws: 1-year post-loading results from a multi-centre randomised controlled trial. Eur. J. Oral Implantol..

[14] Esposito M., Barausse C., Pistilli R., Piattelli M., Di Simone S., Ippolito D.R., Felice P. (2019). Posterior atrophic jaws rehabilitated with prostheses supported by 5 × 5 mm implants with a nanostructured calcium-incorporated titanium surface or by longer implants in augmented bone. Five-year results from a randomised controlled trial. Eur. J. Oral Implantol..

[15] Felice P., Barausse C., Pistilli R., Ippolito D.R., Esposito M. (2019). Five-year results from a randomised controlled trial comparing prostheses supported by 5-mm long implants or by longer implants in augmented bone in posterior atrophic edentulous jaws. Int. J. Oral Implantol..

[16] Felice P., Pistilli R., Barausse C., Piattelli M., Buti J., Esposito M. (2019). Posterior atrophic jaws rehabilitated with prostheses supported by 6-mm-long 4-mm-wide implants or by longer implants in augmented bone. Five-year post-loading results from a within-person randomised controlled trial. Int. J. Oral Implantol..

[17] Esposito M., Barausse C., Pistilli R., Checchi V., Diazzi M., Gatto M.R., Felice P. (2015). Posterior jaws rehabilitated with partial prostheses supported by 4.0 × 4.0 mm or by longer implants: Four-month post-loading data from a randomised controlled trial. Eur. J. Oral Implantol..

[18] Felice P., Barausse C., Pistilli R., Spinato S., Bernardello F. (2014). Guided “Sandwich” Technique: A Novel Surgical Approach for Safe Osteotomies in the Treatment of Vertical Bone Defects in the Posterior Atrophic Mandible: A Case Report. Implant. Dent..

[19] Lamazza L., Garreffa G., Laurito D., Lollobrigida M., Palmieri L., De Biase A. (2016). Temperature Values Variability in Piezoelectric Implant Site Preparation: Differences between Cortical and Corticocancellous Bovine Bone. BioMed Res. Int..

[20] Stefanelli L., De Groot B., Lipton D., Mandelaris G. (2019). Accuracy of a Dynamic Dental Implant Navigation System in a Private Practice. Int. J. Oral Maxillofac. Implants..

[21] Kaewsiri D., Panmekiate S., Subbalekha K., Mattheos N., Pimkhaokham A. (2019). The accuracy of static vs. dynamic computer-assisted implant surgery in single tooth space: A randomized controlled trial. Clin. Oral Implant. Res..

[22] Franchina A., Stefanelli L.V., Gorini S., Fedi S., Lizio G., Pellegrino G. (2020). Digital Approach for the Rehabilitation of the Edentulous Maxilla with Pterygoid and Standard Implants: The Static and Dynamic Computer-Aided Protocols. Methods Protoc..

[23] Pellegrino G., Pavanelli F., Ferri A., Lizio G., Parulli R., Marchetti C. (2020). Ultrasonic Navigation for the Treatment of Medication-Related Jaw Osteonecrosis Involving the Inferior Alveolar Nerve: A Case Report and Protocol Review. Methods Protoc..

[24] Pellegrino G., Taraschi V., Zacchino A., Ferri A., Marchetti C. (2019). Dynamic navigation: A prospective clinical trial to evaluate the accuracy of implant placement. Int. J. Comput. Dent..

[25] Ferrini F., Capparé P., Vinci R., Gherlone E.F., Sannino G. (2018). Digital versus Traditional Workflow for Posterior Maxillary Rehabilitations Supported by One Straight and One Tilted Implant: A 3-Year Prospective Comparative Study. BioMed Res. Int..

[26] Polizzi E., Tetè G., Bova F., Pantaleo G., Gastaldi G., Capparè P., Gherlone E. (2020). Antibacterial properties and side effects of chlorhexidinebased mouthwashes. A prospective, randomized clinical study. J. Osseointegration..

[27] Felice P., Bertacci A., Bonifazi L., Karaban M., Canullo L., Pistilli R., Sammartino P., Gasparro R., Barausse C. (2021). A proposed protocol for ordinary and extraordinary hygienic maintenance in different implant prosthetic scenarios. Appl. Sci..

[28] Parisi M.R., Tecco S., Gastaldi G., Polizzi E., D’Amicantonio T., Negri S., Gardini I., Schlusnus K., Gherlone E., Capparè P. (2017). Point-of-care testing for hepatitis C virus infection at alternative and high-risk sites: An Italian pilot study in a dental clinic. New Microbiol..

[29] Vinci R., Rebaudi A., Capparè P., Gherlone E. (2011). Microcomputed and histologic evaluation of calvarial bone grafts: A pilot study in humans. Int. J. Periodontics Restor. Dent..

[30] Traini T., Piattelli A., Caputi S., Degidi M., Mangano C., Scarano A., Perrotti V., Iezzi G. (2015). Regeneration of human bone using different bone substitute biomaterials. Clin. Implant. Dent. Relat Res..

[31] Sberna M.T., Rizzo G., Zacchi E., Capparè P., Rubinacci A. (2009). A preliminary study of the use of peripheral quantitative computed tomography for investigating root canal anatomy. Int. Endod. J..

